# RPS3A positively regulates the mitochondrial function of human periaortic adipose tissue and is associated with coronary artery diseases

**DOI:** 10.1038/s41421-018-0041-2

**Published:** 2018-08-21

**Authors:** Yan Tang, Yi He, Chen Li, Wenjuan Mu, Ying Zou, Conghui Liu, Shuwen Qian, Fuchuang Zhang, Jiabao Pan, Yina Wang, Haiyan Huang, Dongning Pan, Pengyuan Yang, Ju Mei, Rong Zeng, Qi-qun Tang

**Affiliations:** 1 Key Laboratory of Metabolism and Molecular Medicine of Chinese Ministry of Education, Department of Biochemistry and Molecular Biology of School of Basic Medical Sciences and Department of Endocrinology of Zhongshan Hospital, Fudan University, 200032 Shanghai, China; 2 Department of Cardiothoracic Surgery, Xinhua Hospital, Shanghai Jiaotong University of Medicine College, 200032 Shanghai, China; 3 Key Laboratory of Systems Biology, Institute of Biochemistry and Cell Biology, Shanghai Institutes for Biological Sciences, Chinese Academy of Sciences, Shanghai, China

## Abstract

Pericardial adipose tissue, which comprises both epicardial adipose tissue (EAT) and paracardial adipose tissue (PAT), has recently been recognized as a novel factor in the pathophysiology of cardiovascular diseases, especially coronary artery disease (CAD). The goal of this study was to evaluate differences in the brown-like characteristic and proteome among human EAT, PAT, and subcutaneous adipose tissue (SAT) to identify candidate molecules causing CAD. Uncoupling protein 1 (UCP-1) and other brown-related proteins were highly expressed in pericardial adipose tissue but was weakly expressed in SAT from the same non-CAD patient. Moreover, pericardial adipose tissues displayed a higher thermogenesis than SAT. However, brown-related genes were lower in CAD pericardial fat. Remarkably, there were lower levels of metabolic enzymes involved in glycolysis, tricarboxylic acid cycle, and fatty acid metabolism in pericardial adipose tissues of CAD. EAT is an organ adjacent to aortic root without anatomy barriers, which differs from PAT. We found that the expression of ribosomal protein S3A (RPS3A) was decreased in human EAT as well as in mouse perivascular adipose tissue (PVAT). Knockdown of RPS3A significantly inhibited adipocyte differentiation in preadipocytes and impaired the function of mitochondria in mature adipocytes. Moreover, RPS3A knockdown in mouse periaortic adipose tissue impaired browning of PVAT, accelerated vascular inflammation, and atherosclerosis progression. Mechanistically, RPS3A can migrate to the mitochondria to maintain the function of brown adipocytes. These findings provide compelling evidence that RPS3A was a key factor for modulating the brown fat-specific gene UCP-1 and carbon metabolic enzymes in EAT for preventing CAD.

## Introduction

Obesity is one of the main causes of metabolic syndrome, and is associated with chronic inflammation and cardiovascular disease^1^. However, according to the Framingham Heart Study, metabolic risk factors are more associated with omental adipose tissue than with subcutaneous adipose tissue (SAT)^2^. These differences are plausibly due to differences in adipose tissue distribution and metabolism.

There are two major types of adipose tissue: white adipose tissue (WAT) and brown adipose tissue (BAT). WAT is composed of adipocytes with a large, single fat droplet and is presumed to be the main depot for lipid storage, whereas BAT contains several smaller fat droplets and numerous mitochondria and is involved in heat production^3^. BAT is present throughout life in rodents, whereas in humans, it was thought to rapidly involute postnatally, essentially disappeared within the first years after birth^4^. However, positron emission tomography (PET) and X-ray computed tomography (CT) showed that active uncoupling protein 1 (UCP1)-expressing adipocytes, including brown adipocytes or beige cells, are localized close to the clavicular, periaortic, cervical, and suprarenal regions in adulthood^5,6^. Both brown and beige adipocytes are functionally thermogenic and are considered to be promising new therapeutic avenue to combat atherosclerosis and obesity.

In addition to the classical BAT existing in the interscapular, recent studies have found that perivascular adipose tissue (PVAT) is a vasoactive organ with functional characteristics similar to BAT, and plays an important protective role in the pathogenesis of atherosclerosis^7^. PVAT directly abuts the adventitia of blood vessels and actively communicates with the vascular wall to regulate vascular function and inflammation^8^. Loss of PVAT surrounding the vasculature causes temperature loss and endothelial dysfunction, and promotes atherosclerosis in mice^7^. Other studies have found that cold exposure and beta-3 adrenergic receptor (β3-AR)-mediated BAT activation enhances the selective uptake of fatty acids from triglyceride (TG)-rich lipoproteins into BAT, subsequently accelerating the hepatic clearance of the cholesterol-enriched remnants and protecting from atherosclerosis^9^.

In humans, epicardial adipose tissue (EAT) predominantly functions as PVAT for the coronary arteries, and displays high rates of both lipogenesis and lipolysis. It is thought to serve as a local fat storage depot, storing excess free fatty acids as TG at times of excess and releasing them to the heart for substrate utilization in times of metabolic stress^10^. In addition, paracardial adipose tissue (PAT) is the fat surrounding the parietal pericardium, termed “mediastinal fat” or “thoracic fat”. Recent data in humans suggest that EAT and PAT may be “beige” in morphology, with features of both WAT and BAT^11–13^. Measurements of EAT and PAT volume have suggested that the thickness of pericardial adipose tissue, which comprises both EAT and PAT, may serve as a risk factor and biomarker to predict the early stages of atherosclerosis and coronary heart disease (CAD)^14–16^. Given the different depot-specific gene expression profiles in “brown” adipocytes, the purpose of this study was to provide a better understanding of the putative thermogenic function of human EAT and PAT using tissues obtained from patients undergoing open heart surgery with and without CAD.

Locally produced adipokines by pericardial adipose tissue might reflect or affect cardiovascular pathology due to its proximity to coronary arteries^14^. We hypothesized that there are differences in the proteome patterns in pericardial adipose tissue from patients with and without CAD. Among the proteins, ribosomal protein S3A (RPS3A) exhibited 3-fold lower expression in EAT from CAD patients. RPS3A, a component of the ribosomal small subunit (40S), shows high sequence homology in mammalian cells and is localized in both the nucleus and cytoplasm^17^. RPS3A also has multiple biological functions unrelated with the ribosome. Previously known as v-fos transformation effector gene, RPS3A is an inducing factor for the oncogenic cellular transformation of Rat-1 fibroblasts by v-fos^18^. In addition, RPS3A is highly expressed in most tumors^19,20^. However, the mechanism underlying the role of RPS3Acin thermogenesis remains unknown.

In this study, we found that in non-CAD patients, UCP1 was relatively abundant in EAT and PAT, both depots possess smaller lipid size characteristic of those found in vitro in beige lineage adipocytes. The browning and thermogenesis of EAT and PAT were significantly decreased in patients with CAD. The decreased expression of RPS3A in EAT was associated with a “brown to white” change in mature adipocytes and vascular dysfunction. These results may shed light on the role of pericardial adipose tissue in the physiopathology of atherosclerosis and CAD.

## Results

### Human pericardial adipose tissue displays some characteristics of BAT compared with the subcutaneous depot

As shown in Supplementary Table S1, a total of 58 CAD patients were the case group, and 64 non-CAD patients undergoing valvular heart surgery without CAD were the control group. The CAD and control groups were comparable among several covariates including age, gender, body mass index (BMI), waist size, smoking status, and metabolic syndrome. Individuals with CAD were older than those without CAD, and women had a lower incidence of developing CAD. In addition, waist size was significantly higher, whereas BMI was similar between CAD patients and the control group. CAD patients were more likely to have a history of hypertension. However, history of diabetes mellitus, serum TG, total cholesterol (TC), high-density lipoprotein (HDL), and low-density lipoprotein (LDL) were not significantly different between CAD patients and controls (all *p* > 0.05), which may be explained by the older population. From these characteristics and laboratory findings, we hypothesized that other factors may modulate atherosclerosis besides classical factors.

**Fig. 1:**
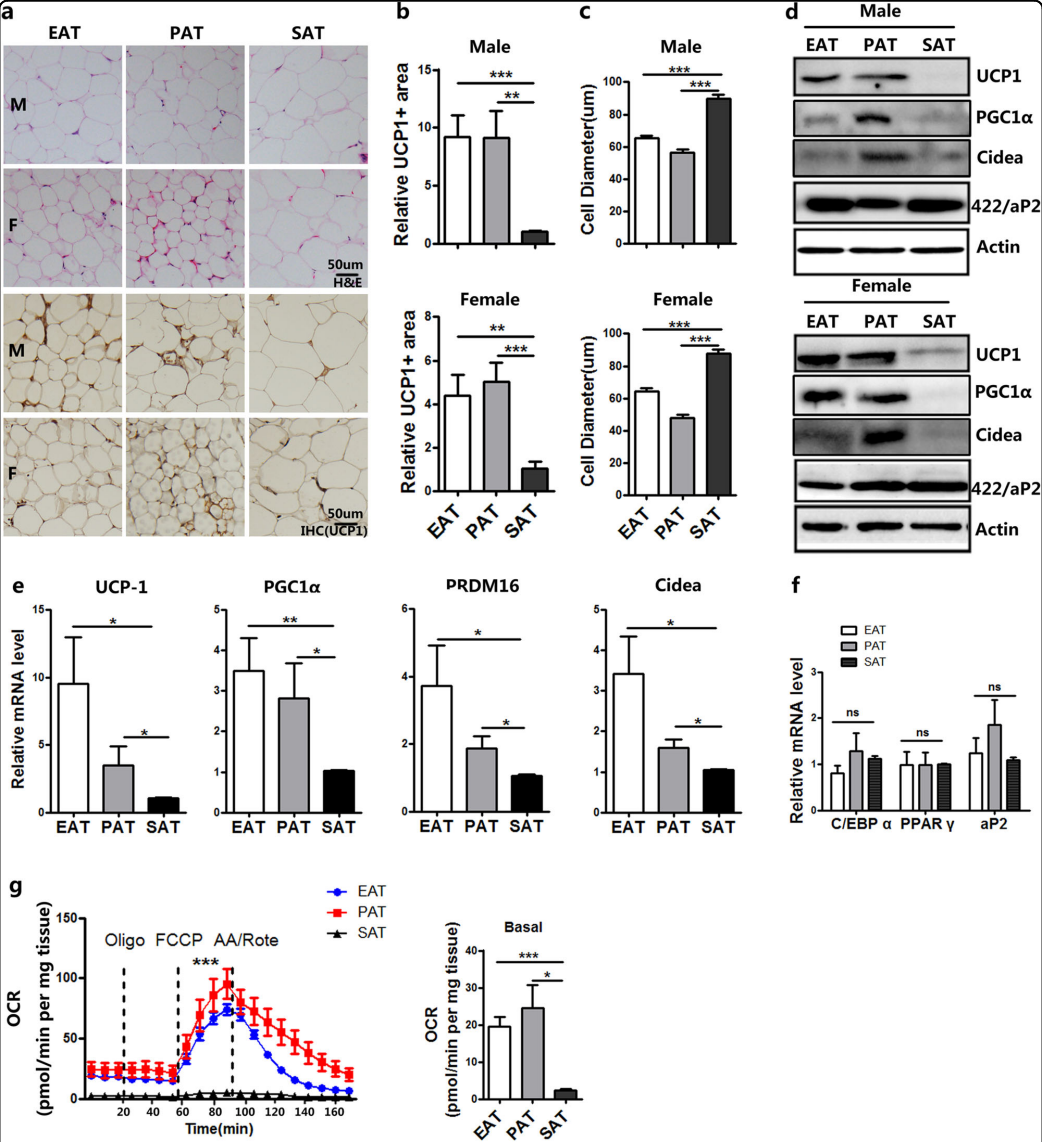
Differential phenotype between epicardial, paracardial, and subcutaneous adipose tissue. **a** Representative HE and IHC (UCP-1) microscopic picture of epicardial, paracardial, and subcutaneous adipose tissue (EAT, PAT, and SAT) samples from one patient. M male, F female. **b** Quantification of UCP-1^+^ area of EAT, PAT, and SAT (data were collected using Image Pro-Plus software for IHC analysis of three individual patients, three fields per person). **c** Quantification of adipocyte diameter of EAT, PAT, and SAT from the same patients without CAD (data were collected by using Image J software from H and E staining section of three individual patients, 8–12 fields per patient, 24–35 cells per field in each group). **d** Western blot experiment using antibodies against UCP1, PGC1α, Cidea, Ap2, and Actin of EAT, PAT, and SAT from the same patient. **e** Real-time PCR data showing the fold induction of indicated brown adipocyte related genes with expression normalized to the housekeeping gene 18s in EAT, PAT, and SAT from patients without CAD (*n* = 10–15). **f** Real-time PCR data showing the fold induction of indicated white adipocyte-related genes with expression normalized to the housekeeping gene 18s in EAT, PAT, and SAT from patients without CAD (*n* = 9–11). **g** OCR in adipose tissues derived from EAT, PAT, or SAT. Oligomycin (oligo), FCCP, and antimycin/rotenone (AA/Rote) were added at the time points indicated by dashed lines (left), and averaged basal OCR is shown (right) (*n* = 4)

EAT, PAT, and SAT were obtained from the same subjects undergoing heart surgery without CAD. Hematoxylin and eosin (H&E) staining and immunohistochemistry (IHC) showed that the tissue morphologies were generally similar in the three depots (Fig. 1a). However, the cross-sectional area of adipocytes in the EAT and PAT were significantly less than that in SAT (Fig. 1c). Multilocular lipid droplets were observed in some adipocytes in PAT from female patients, whereas only unilocular lipid droplets were observed in SAT adipocytes from the same patients (Fig. 1a and Supplementary Fig. S1a). Regions of EAT and PAT were stained positively for UCP1, but weak or no staining for UCP1 was observed in SAT (Fig. 1a, b). The protein expressions of UCP1, PGC1α and Cidea were measured by western blotting and found to be relatively high in pericardial adipose tissue, whereas the expression level of the WAT marker aP2 was the same (Fig. 1d). qPCR analysis revealed a significantly higher expression of UCP1, PGC1α, PRDM16, and Cidea in the pericardial adipose tissue compared to the subcutaneous depot, but a similar expression of C/EBPα, PPARγ, aP2 (Fig. 1e, f). We also found that the oxygen consumption rate (OCR) of pericardial adipose tissues were significantly higher than SAT (Fig. 1g).

To further verify the brown-like characteristics of pericardial adipose tissue, stromal vascular fraction (SVF) was isolated from adipose tissues and differentiated into mature adipocytes in vitro. Primary SVF derived from EAT, PAT, and SAT was cultured and differentiated toward beige adipocytes. SVF from PAT and SAT showed higher adipogenic ability compared with EAT (Supplementary Fig. S2a). Other studies have shown that beige adipocytes have smaller lipid droplet; we evaluated morphological changes using Oil Red O staining. Adipocytes differentiated from PAT-SVF demonstrated the reduced size of lipid droplets, concomitantly the increased protein level of UCP1 and the mRNA levels of UCP1, PGC1α, and PRDM16 (Supplementary Fig. S2b–e). These results provided strong evidence confirming the brown-like characteristics of pericardial adipose tissue.

### Metabolic disorders in EAT and PAT from CAD patients

**Fig. 2:**
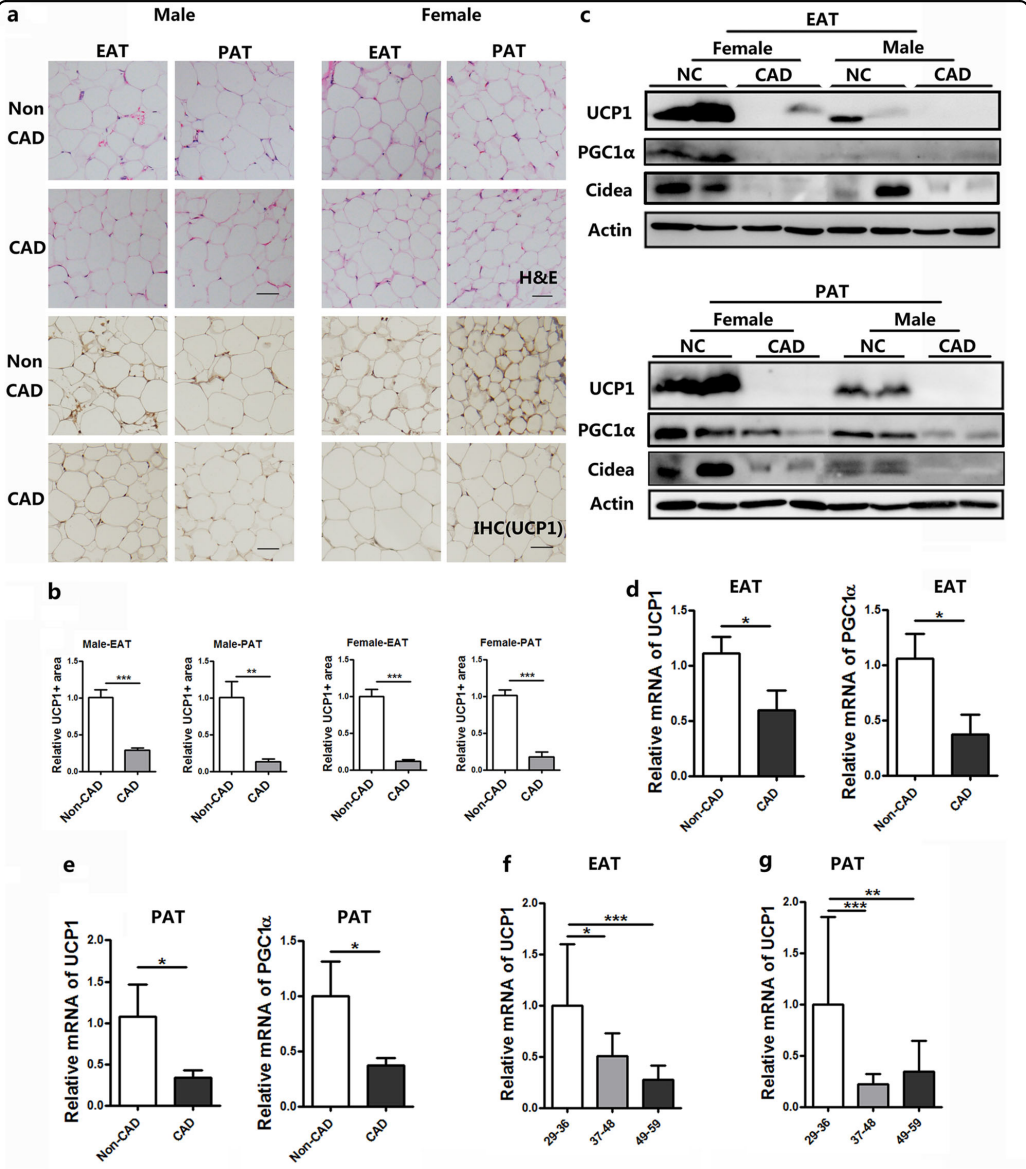
UCP1 expressions in human pericardial adipose tissue from patients with vs. without CAD. **a** Representative HE microscopic picture and immunohistochemical staining of UCP1 of epicardial and paracardial adipose tissue samples from patient with or without CAD. **b** Quantification of UCP-1^+^ area of EAT and PAT from patients with or without CAD. (Data were collected using Image Pro-Plus software for IHC analysis of three individual patients, three fields per person.) **c**, **d** Western blot experiment using antibodies against UCP1, PGC1α, Cidea, and Actin of EAT and PAT from patients with or without CAD. **e**, **f** Real-time PCR data showing the fold induction of indicated brown adipocyte-related genes with expression normalized to the housekeeping gene 18s in EAT and PAT from patients with or without CAD (*n* = 12–16). **g**, **h** Real-time PCR data showing the fold induction of UCP1 with expression normalized to the housekeeping gene 18s in EAT and PAT from patients with different level of CAD (*n* = 8–13)

Having identified the brown-like characteristic of pericardial adipose tissue, we reasoned that the browning of pericardial adipocyte may be decreased in CAD patients. Indeed, UCP1 staining of pericardial adipocytes from CAD patients showed much less brown staining than non-CAD patients (Fig. 2a, b). Changes were further confirmed by the decreased mRNA levels of UCP1 and PGC1α (Fig. 2e, f) and diminished UCP1, PGC1α, and Cidea protein expressions in CAD patients (Fig. 2c and d). However, the expressions of UCP1 and PGC1α were the same in non-pathological epicardial adipose tissue (EAT-NC) from CAD and non-CAD patients (Supplementary Fig. S3a). All CAD patients had SYNTAX score II by applying the Cox proportional hazards model. These CAD patients were divided into three groups and the mRNA levels of UCP1 and PGC1α were detected. Patients with the highest SYNTAX score had a lower expression of UCP1 in both EAT and PAT (Fig. 2g, h). These data indicate a “brown-to-white” shift of pericardial adipose tissue in CAD patients.

**Fig. 3:**
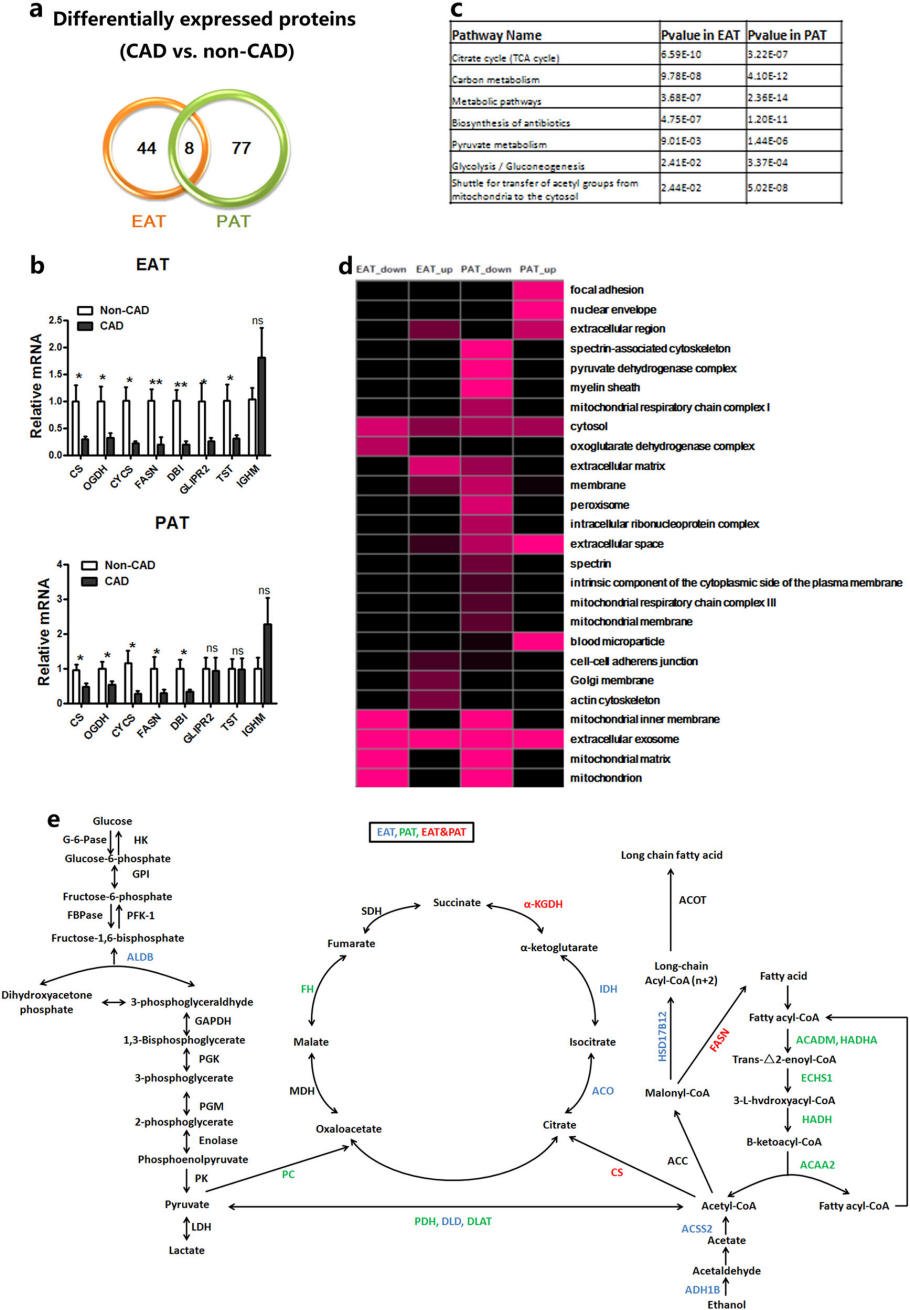
Heat map showing the expression of the literature-curated mitochondrial function genes in the epicardial and pericardial adipose tissue of patients with vs. without CAD in the RYGB profiling experiments. **a** Venn map of the differentially expressed proteins found in EAT or PAT tissues comparing CAD and non-CAD cases. **b** Real-time PCR validation of genes selected from the proteomic analysis (*n* = 8–12). **c** Enriched metabolic-associated KEGG pathways with downregulated expressed proteins in EAT or PAT tissues were shown by table. **d** Enriched GO-CC terms (hypergeometric test, *p* value < 0.05) in down- or upregulated expressed proteins of EAT or PAT between CAD and non-CAD cases. Gradations of purple stand for the significant degrees (−log (*p* value, 10)) of enrichment. **e** Proteins identified changed in CAD samples by proteomic survey are marked in blue (only EAT), green (only PAT), and red (EAT and PAT)

To describe the systemic regulations and find useful biomarkers of EAT and PAT in CAD patients, the EAT or PAT proteome with or without CAD were analyzed by tandem liquid chromatography-tandem mass spectrometry (LC-MS/MS) using a label-free strategy. From 10 pairs patients, there were a total of 52 proteins from EAT, and 85 proteins from PAT that were differentially expressed between CAD and non-CAD patients (Fig. 3a). Comparison of the two data sets indicated that there were eight proteins overlapped in both EAT and PAT, which was confirmed by qPCR analysis (Fig. 3b). In order to give a proteomic snapshot, we outlined the changed proteome and discovered that enzymes participating in intermediate metabolism were preferentially changed in EAT and/or PAT of CAD (Fig. 3c), which agrees with that of the previous researches^21^. More interestingly, most of the changed proteins were concentrated in the mitochondria (Fig. 3d), illustrating the mitochondrial oxidative stress play an important role in CAD^22,23^. Indeed, most of the enzymes involved in glycolysis, the tricarboxylic acid (TCA) cycle, and fatty acid metabolism showed a decreased trend in CAD patients (Fig. 3e). These results uncover a previously unrecognized and potentially extensive role of pericardial adipocyte metabolism in regulation of atherosclerosis.

### RPS3A is required for brown adipogenesis to maintain epicardial adipocyte dynamics

**Fig. 4:**
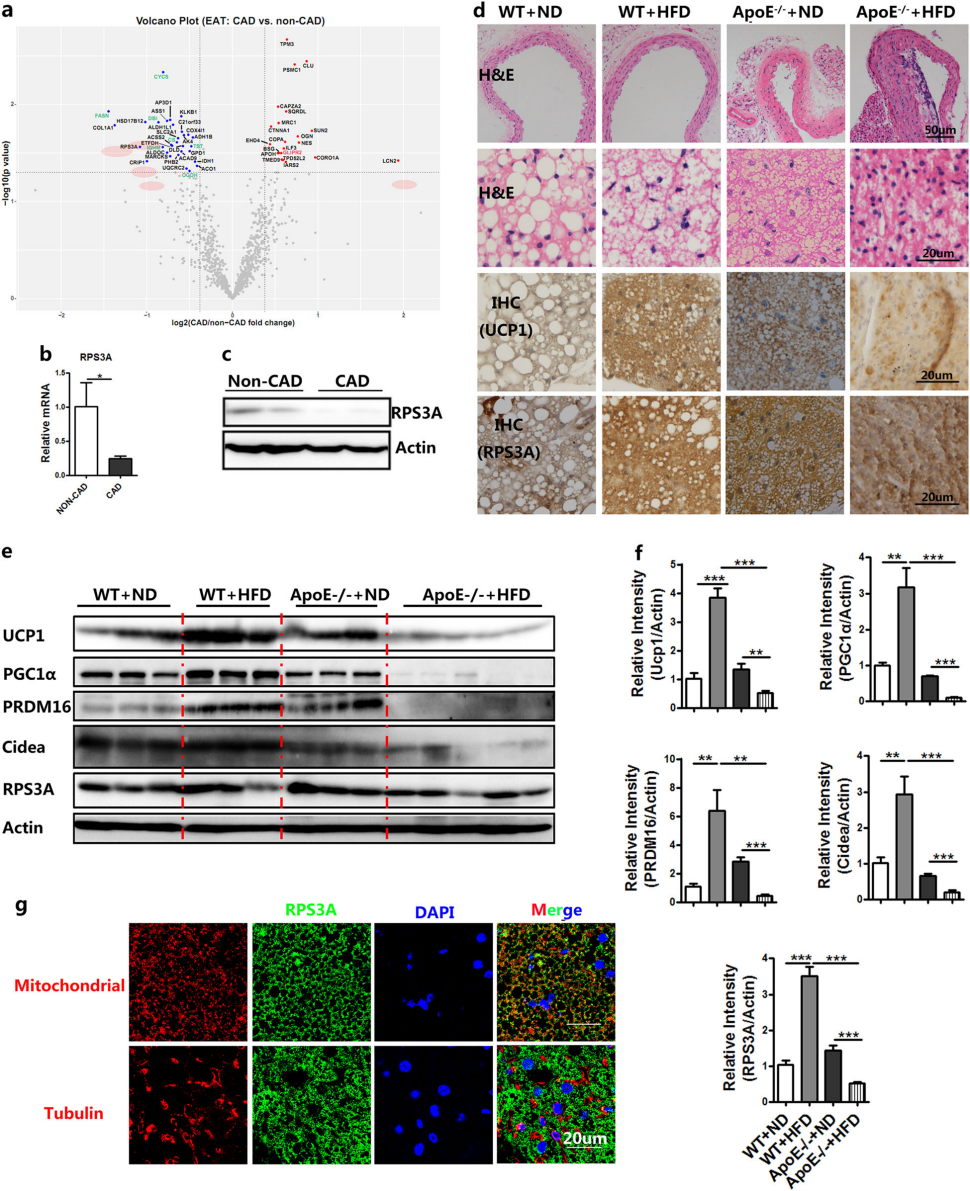
The expression of RPS3A is decreased in perivascular adipose tissue to inhibit stem cell adipogenesis. **a** Volcano plot of EAT proteome comparing CAD and non-CAD patients. The gene symbols of 31 downregulated proteins and 21 upregulated proteins in CAD cases were marked. **b** Real-time PCR validation of RPS3A from the EAT proteomic analysis of patients with or without CAD (*n* = 7–9). **c** Western blot experiment using antibodies against RPS3A and Actin of EAT from patients with or without CAD. **d** H&E staining and Immunohistochemical staining of UCP1, RPS3A of vascular (top), and adipose tissue (middle and bottom) sections obtained from 8-week-old WT littermate control mice fed normal chow diet (WT + ND), 8-week-old WT littermate control mice fed a high-fat diet (WT + HFD), ApoE^−/−^ mice fed normal chow diet (ApoE^−/−^ + ND), and ApoE^−/−^ mice fed a high-fat diet (ApoE^−/−^ + HFD) for 4 months. **e** Western blot experiment using antibodies against UCP1, PGC1α, PRDM16, Cidea, RPS3A, and Actin of PVAT from mice as indicated above. **f** Relative gray intensity of the band (E) was quantitated using Image J software (*n* = 6). **g** Confocal microscopic images of PVAT sections immunostained for mitochondria/tubulin (red), RPS3A (green) and DAPI (blue)

To determine the mechanism underlying the “brown to white” changes of pericardial adipose tissue, we next identified the potential candidate proteins in EAT, which is derived from aortic root. In the clinic, the presence of aortic atherosclerosis can be used as an additional marker for predicting CAD^24^. In our study, we also found calcified plaque around the aortic root in CAD compared with non-CAD patients (Supplementary Fig. S3b). First, we examined the expression of adrenergic receptor beta (ARβ), thyroid hormone receptor beta (ThRβ), M1 macrophage-related gene (TNFα), and M2 macrophage-related gene (Mrc1) in EAT from non-CAD and CAD patients. However, there were no significant changes with exception of the inflammation-related gene TNFα (Supplementary Fig. S3c). Second, based on our proteomic data, the abnormal regulation of the metabolic pathway was shown in CAD tissues (Fig. 4a). The five proteins, Col1a1, RPS3A, HSD17B12, Crip1, and LCN2, which were changed specially in EAT (Fig. 4a, Supplementary Fig. S3d). Given the multifunction of ribosome protein, we determined its functional role during brown adipocyte differentiation. Real-time PCR and western blotting analysis confirmed that RPS3A was significantly decreased in CAD tissue (Fig. 4b, c).

ApoE^−/−^ mice have been widely used as an atherosclerosis animal model. Thus, we next investigated RPS3A expression level in PVAT of WT fed ND (normal diet), WT fed HFD (high-fat diet), ApoE^−/−^ fed ND, and ApoE^−/−^ mice fed HFD for 4 months. We found that HFD feeding induced WT mice browning process in PVAT, which was associated with an increased expression of RPS3A. Importantly, ApoE^−/−^ atherosclerosis mice had decreased expression of UCP1, PGC1α, PRDM16, Cidea, and RPS3A (Fig. 4d–f). Confocal microscopic analysis was conducted to demonstrate the tissue location of RPS3A. Colocalization between RPS3A and mitochondria was detected, while colocalization between RPS3A and tubulin was not exist (Fig. 4g).

**Fig. 5:**
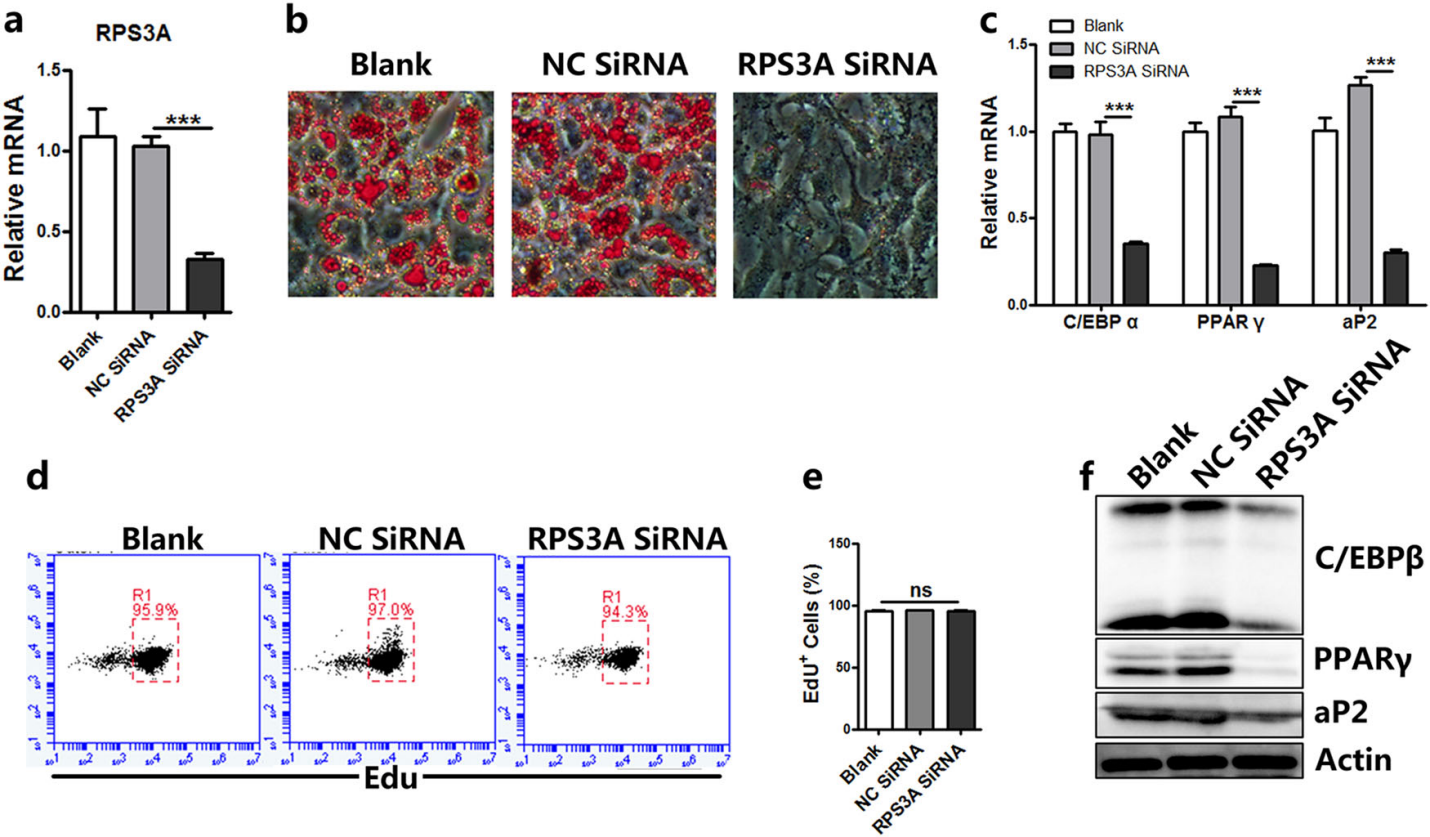
RPS3A knockdown in brown preadipocyte suppresses adipogenesis. **a** Brown preadipocytes were treated with RNAi before adipogenic induction and collected for qPCR to determine relative mRNA expression levels of RPS3A (*n* = 3). **b** Oil Red O staining of adipocytes differentiated from brown preadipocytes on day 8. **c** qPCR to determine the decreased mRNA expression of adipocyte markers (*n* = 3). **d** and **e** Flow cytometric analysis of EdU+ cells in brown preadipocytes (*n* = 3). **f** Brown preadipocytes were treated with RNAi before adipogenic induction and Western blot experiment using antibodies against C/EBPβ, PPARγ, aP2, and Actin on day 4

To further explore the function of RPS3A in the process of adipogenesis, specific Stealth^TM^ RNAi of RPS3A was used to knockdown its expression in immortalized brown preadipocytes (Fig. 5a). Knocking-down RPS3A inhibited the adipogenic ability and decreased the expression of adiocyte-specific markers (Fig. 5b, c). Further investigations showed a decreased expression of RPS3A in 3T3-L1 and C3H10T1/2 cell line both inhibited adipogenesis (Supplementary Fig. S4a–f). Previous findings indicate that the preadipocyte will first undergo mitotic clonal expansion (MCE) and subsequently terminal differentiation during the adipogenesis, while C/EBPβ is required for both of these events^25^. The RNAi effect of RPS3A on DNA synthesis was evaluated by using EdU labeling. As shown in Fig. 5d, e, RPS3A knockdown did not cause less EdU incorporation. Adipogenesis is controlled by a transcriptional cascade composed of a large number of transcriptional factors, including CCAAT/enhancer-binding proteins (C/EBPs), peroxiaome proliferator-activated receptor γ (PPARγ), signal transducers and activators of transcription (STATs), and Kruppel-like factor (KLF) proteins^26^. C/EBPβ is induced very early in adipocyte differentiation. Then it activates the expression of C/EBPα and PPARγ, two critical pro-adipogenic transcription factors, by binding to their promoters^27^. Previous studies have shown that knockdown of C/EBPβ and PPARγ prevent adipocyte differentiation^28,29^. We found that downregulation of RPS3A reduced the expression of C/EBPβ, and subsequent PPARγ and 422/aP2 proteins (Fig. 5f). Collectively, these results indicated that RPS3A may play a dominant role in preadipocyte differentiation.

### RPS3A promotes the function of mature brown adipocytes to inhibit atherosclerosis

**Fig. 6:**
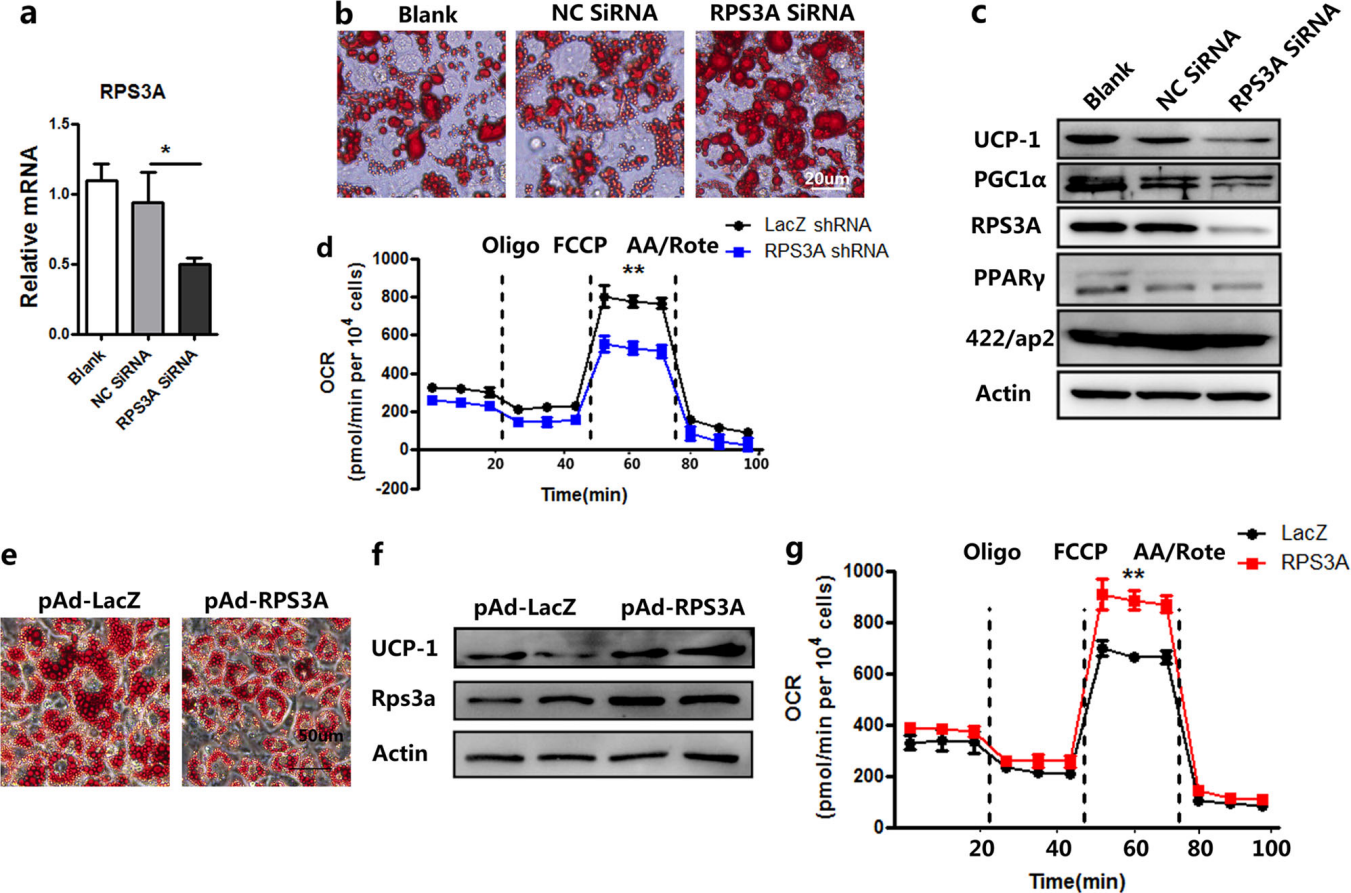
RPS3A knockdown suppresses the function of mature brown adipocyte. On day 4 after adipogenic induction, RPS3A was silenced by siRPS3A. The indicated cells were then harvested on day 8 post induction and subjected to further studies. **a** qPCR to determine relative mRNA expression levels of RPS3A (*n* = 3). **b** Oil Red O staining of adipocytes differentiated from brown preadipocytes on day 8. **c** Western blotting was conducted to measure the level of the indicated brown and white adipocyte related proteins. **d** OCR in brown adipocytes treated with Ad-LacZ shRNA or Ad-RPS3A shRNA. Oligomycin (oligo), FCCP, and antimycin/rotenone (AA/Rote) were added at the time points indicated by dashed lines (*n* = 3). **e** On day 5 after white adipogenic induction, adipocytes were infected with Ad-RPS3A. On day 8, Oil Red O staining of adipocytes was used to observe the size of lipid droplet. **f** Indicated cells were harvested, and Western blotting was conducted to measure the level of the indicated proteins. **g** OCR in mature adipocytes treated with Ad-LacZ or Ad-RPS3A. Oligomycin (oligo), FCCP, and antimycin/rotenone (AA/Rote) were added at the time points indicated by dashed lines (*n* = 3)

Knockdown of RPS3A in preadipocytes inhibited adipogenesis, so as to suppress more stem cell differentiation into mature adipocytes in the physiological or pathological state, which is consistent with clinical studies that HIV-related lipodystrophy syndrome is associated with an increased risk for the development of cardiovascular disease^30^. On the other hand, clinical studies have found that a larger pericardial fat volume is associated with a higher risk of CAD. We hypothesized that RPS3A might be a potential regulator for maintaining the function of brown adipocytes. To test this presumption, we examined the effects of RPS3A knockdown or overexpression on the brown adipocytes by performing RNA interference for RPS3A (SiRPS3A) or infecting adipocytes with Ad-RPS3A in mature adipocytes. As expected, RPS3A mRNA and protein expression were significantly reduced by SiRPS3A (Fig. 6a, c). Oil Red O staining showed that knockdown of RPS3A increased lipid droplet size (Fig. 6b). In addition, the protein expression of UCP1 and PGC1α was significantly reduced by siRPS3A (Fig. 6c). Furthermore, knockdown of RPS3A decreased OCR in brown adipocytes (Fig. 6d). In contrast, overexpression of RPS3A by Ad-RPS3A increased small multiple-lipid droplet size (Fig. 6e) and effectively rescued the UCP1 expression and OCR level of brown adipocyte caused by white adipocyte induction cocktail without indometacin and T3 (Fig. 6f, g).

**Fig. 7:**
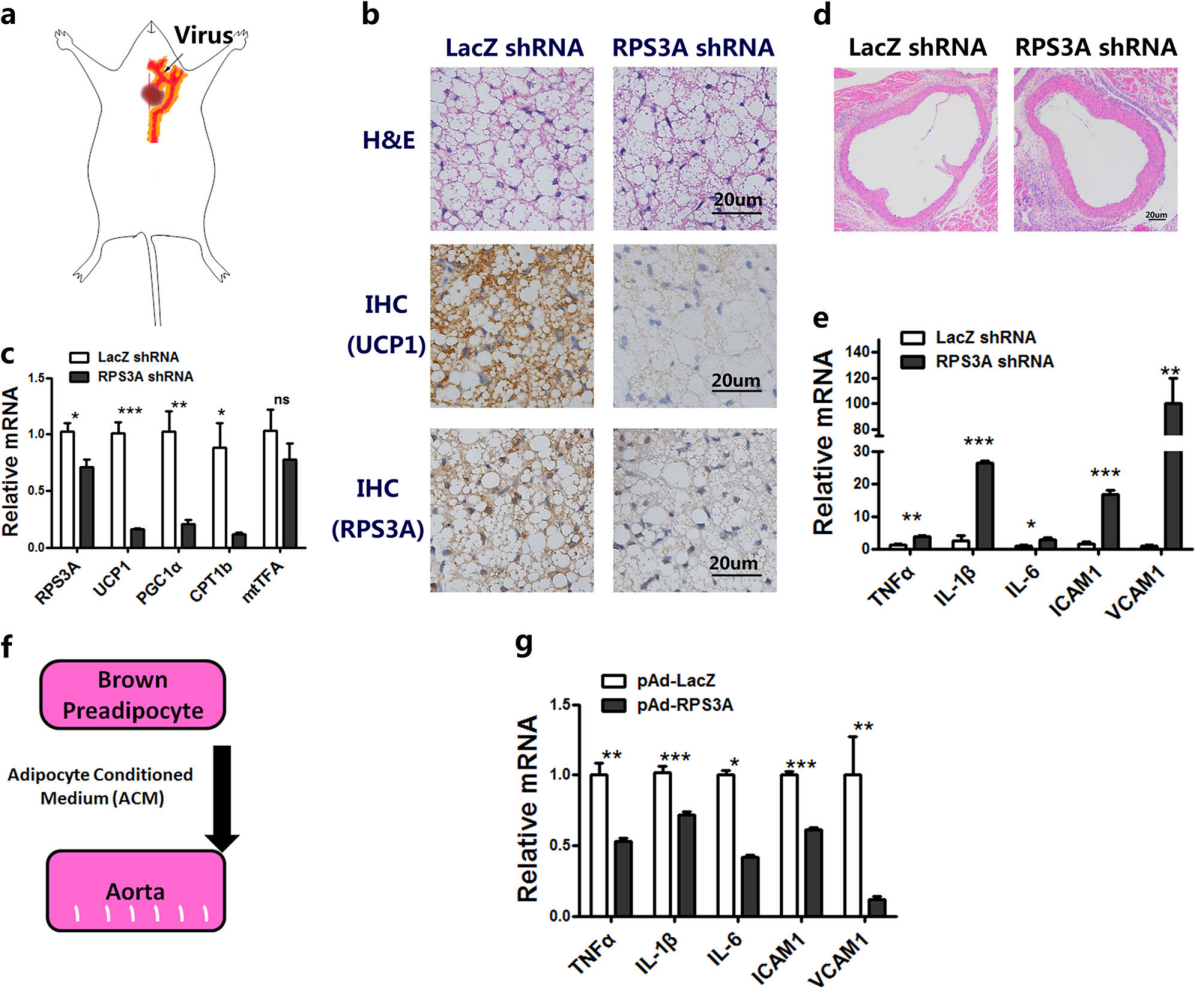
RPS3A-regulated PVAT plays an important role in atherosclerosis in vivo. **a** Images of mouse cardiovascular following injection of adenovirus adjacent to the periaortic arch. **b**–**e** Adenovirus expressing RPS3A shRNA was injected weekly adjacent to the aortic arch for 2 weeks. LacZ shRNA was simultaneously injected as a control. **b** Periaortic arch adipose tissues were subjected to H&E staining and immunohistochemical staining with UCP1, RPS3A. **c** qPCR of mRNAs encoding brown adipocyte markers in the periaortic arch adipose tissue (*n* = 4). **d** Representative sections of aortic arch stained with H&E. **e** qPCR of mRNAs encoding inflammation and macrophage markers in the aorta arch (*n* = 4). **f** Adipocyte-conditioned medium was cultured with aorta from mice. **g** The aortic sections from control mice were incubated with CM derived from mature adipocytes, and treated with pAd-LacZ or pAd-RPS3A for 24 h; the aortic sections were harvested and qPCR of mRNAs encoding inflammation and macrophage markers were analyzed (*n* = 3)

To gain deeper insights into the importance of RPS3A in PVAT to vascular function, we next injected adenovirus LacZ shRNA or RPS3A shRNA in the PVAT around the aortic arch (Fig. 7a). Knockdown of RPS3A decreased the activation of PVAT as evidenced by reduced UCP1 protein content per area and reduced thermogenic genes (Fig. 7b, c). In addition, we also depleted RPS3A in inguinal adipose tissue of WT mice by infecting Ad-shRPS3A into the left SAT, while Ad-shLacZ was injected into the right SAT of the same mice. Injection of RPS3A shRNA into SAT resulted in larger size of adipocytes from H&E staining of adipose tissues (Supplementary Fig. S5a). UCP1 protein content per area of inguinal adipose tissue was decresed injected with RPS3A shRNA, suggesting the inactivation of beige adipose tissue (Supplementary Fig. S5a). Moreover, knockdown of RPS3A caused a decreased expression of UCP-1, PGC1α, PRDM16, and Cidea (Supplementary Fig. S5b). All these results indicated that RPS3A knockdown decreased the browning process in SAT during cold exposure. In line with the results obtained from human samples, inhibition of RPS3A in the PVAT increased the thickness of the vasculature and contributed to vascular inflammation by increasing the levels of TNFα, interleukin 1 beta (IL-1β), IL-6, interleukin adhesion molecule 1 (ICAM1), and vascular cell adhesion molecular 1 (VCAM1) (Fig. 7d, e). BAT could secrete factors that act locally and systemically to influence fuel and energy metabolism^31^. Adiponectin secreted by adipocytes could act on the blood vessels to inhibit neointima formation and macrophage inflammation, and suppressing the hepatic expression of the transcription factor SREBP2, thereby leading to reduced cholesterol synthesis and attenuation of hypercholesterolemia^32^. Interestingly, treatment of aortic vascular with adipocyte-derived conditioned medium (CM) pretreated with adenovirus RPS3A significantly decreased the mRNA expression levels of TNFα, IL-1β, IL-6, ICAM1, and VCAM1 (Fig. 7f, g), and demonstrated that overexpression of RPS3A in brown adipocytes inhibited inflammation of vascular, thereby protecting from atherosclerosis.

### RPS3A migrates to the mitochondria to maintain the function of mitochondria in brown adipocytes

**Fig. 8:**
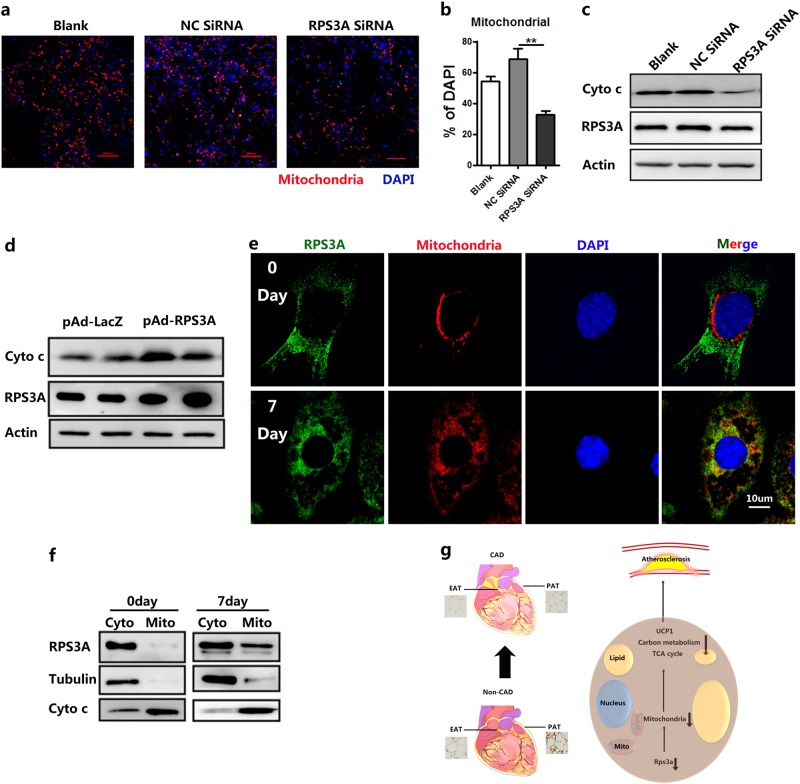
RPS3A is migrated into mitochondria to play a positive role. On day 4 after adipogenic induction, RPS3A was silenced by siRPS3A. The indicated cells were then harvested on day 8 post induction and subjected to further studies. **a** Confocal microscopic images of mature brown adipocytes immunostained for mitochondria (red) and DAPI (blue). **b** Values are the percentage of mitochondria^+^ to DAPI^+^ cells (*n* = 3). **c** Western blotting was conducted to measure the level of the indicated proteins. **d** Mature brown adipocytes were treated with pAd-lacZ/pAd-RPS3A on day 4 after adipogenic induction and collected for Western blotting to determine the indicated proteins. **e** Confocal microscopic images of brown preadipocytes at different time points during differentiation immunostained for mitochondria (red), RPS3A (green), and DAPI (blue). **f** Cells were separated into cytosol and mitochondrial fractions, tubulin was used as marker for the cytosol, cytochrome C was used as a mitochondrial marker. **g** Schematic model illustrates the role of pericardial adipose tissue in atherosclerosis and CAD. The browning of pericardial adipose tissue (both EAT and PAT) plays an important role in maintaining the function of vascular and blood flow. The decreasing expression of RPS3A promotes the disorder of mitochondria and metabolism from periaortic adipose tissue (EAT), which contributes to atherosclerosis and CAD

BAT dissipates energy in the form of heat, which relies on a high abundance of mitochondria as well as high levels of electron transport chain complexes and UCP1 within the mitochondria. Brown preadipocytes were induced and then developed into mature adipocytes with NC SiRNA or RPS3ASiRNA. Knockdown of RPS3A decreased mitochondria biosynthesis as shown by mitochondrial immunofluorescence (IF) staining (Fig. 8a, b). To further verify the effect of RPS3A in mitochondria, we detected the expression of cytochrome C in mature adipocytes, and found that RPS3A knockdown decreased the protein level of cytochrome C, whereas RPS3A overexpression increased its expression (Fig. 8c, d). Next, we investigated the subcellular localization of RPS3A protein by confocal IF analysis using RPS3A and mitochondria antibodies. Interestingly, RPS3A and mitochondria protein expression overlapped in the mature brown adipocytes, whereas RPS3A protein accumulated in the cytosol of the pre-adipocytes (Fig. 8e). To future confirm the mitochondrial translocation of RPS3A, we performed a cytosol/mitochondria fractionation assay. As shown in Fig. 6f, the mitochondrial translocation of RPS3A distinctly increased in the process of brown adipogenesis compared with unstimulated cells.

## Discussion

BAT is essential for adaptive thermogenesis and energy expenditure in human infants and then disappears in adults^33,34^. However, there is accumulating evidence demonstrating that BAT, which is present in areas close to the clavicular, periaortic, cervical, and suprarenal regions, is activated in response to cold exposure^4,6^. In addition, recent studies have shown that PVAT, similar to BAT, is a heat-generating organ that is critical for the maintenance of intravascular temperature and vascular homeostasis^7^. Activation of BAT in hypertriglyceridemic mice by cold strongly reduces plasma TG, illustrating the importance of BAT in lipoprotein metabolism, which is a major risk factor for atherosclerosis^35^. BAT itself is responsible for the uptake of TG-derived fatty acids, leading to the formation of cholesterol-enriched lipoprotein remnants, which are subsequently cleared from the plasma by the liver via binding the ApoE to the hepatic LDL receptor^36^. In humans, daily cold exposure for 20 min for 90 days reduces TC and LDL-cholesterol in hypercholesterolemic individuals^37^. However, there is no such study to compare the browning characteristics between the pericardial adipose tissue and SAT from the same patient without CAD. We are the first to report that pericardial adipose tissue of EAT and PAT exhibits brown cell features while simultaneously expressing high levels of some genes typifying brown adipocytes. Previous work in rodents indicated that BAT is greater in females than males^38^. In our study, we demonstrated that multilocular lipid droplets exist in 50% of female PAT from histology analysis (Supplementary Fig. S1). Using western blot analysis, we present evidence that UCP1 expression was higher in pericardial adipose tissue from females than males (Fig. 2c, d). As a result, there is more browning of pericardial adipose tissue in non-CAD patients, especially in the PAT from female.

The underlying pathological process of CAD is atherosclerosis, which was previously considered a cholesterol deposition disease, and was associated with hypertension, smoking, diabetes, and obesity^39,40^. However, our findings demonstrated that only age, sex, waist size, and hypertension were strongly associated with CAD, in the context of patients older than 55, which prompted us to explore other mechanisms of CAD. In the last decade, BAT has been considered a promising therapeutic target for obesity and associated metabolic disorders^41,42^. The anti-obesity potential of BAT has been irrefutably proven in murine studies and shown in human studies^43,44^. Moreover, BAT activation is beneficial and not deleterious for plasma cholesterol metabolism and atherosclerosis development^9^. However, the effect of BAT activation in human pericardial adipose on atherosclerosis and its relationship to CAD remains controversial to date. Here, we present evidence that BAT activation in pericardial adipose tissue was decreased in CAD patients. Using the SYNTAX score method, we identified that patients with the highest SYNTAX score had lower expression of UCP1, which highlights strong association of UCP1 expression in pericardial adipose tissue with vascular function. In addition, many retrospective analyses have shown that men account for a significantly greater proportion of CAD cases compared with women^45^. This is consistent with our study, in which the ratio of CAD was 58.8% among males and only 33.3% among females. The potential mechanism may be explained by BAT activation in females.

Mitochondria are cytoplasmic organelles in human and animal cells where many distinct metabolic processes take place, including TCA cycle, pyruvate decarboxylation, oxidative decarboxylation of fatty acids (β-oxidation), and degradation of branched amino acids^46^. In addition, specific functions performed by brown fat convert mitochondrial energy into heat in adaptive thermogenesis^47^. Many rodents and human studies have found that obesity and insulin resistance is strongly associated with decreased mitochondria contents in brown or beige adipocytes^48–50^. Moreover, other studies also found a decrease in TCA cycle and β-oxidation gene signatures and an increase in inflammation signatures in the insulin-resistant state, while these gene expression changes were reversed after TZD treatment^51,52^. Here, our findings demonstrated that genes related to the citric acid cycle were under-expressed in the pericardial adipose tissue from CAD patients. Our findings are consistent with a published report showing that abnormal glucose metabolism is a core aspect of metabolic syndrome associated with high risk for heart disease^21^.

EAT is the fat derived from aortic root, while PAT is the fat surrounding the parietal pericardium. In the clinic, the presence of aortic atherosclerosis can be used as an additional marker for predicting CAD. Based on these facts, we analyze the proteins that were changed specifically in EAT. From our proteomic data, five proteins, Col1a1, RPS3A, HSD17B12, Crip1, and LCN2, were changed specially in EAT (Supplementary Fig. S3d and e). We used real-time PCR to validate the five candidates in EAT from non-CAD and CAD patients. RPS3A is obviously decreased in the CAD patients. Given the multifunction of ribosome protein, we chose RPS3A to explore its functional role during brown adipocyte differentiation. RPS3A is a component of the 40S ribosome small subunit and plays a critical role in cell transformation, growth, and many cancers^53^. Over the past decade, more than a dozen ribosomal proteins have been shown to be involved in various physiological and pathological processes^54^. RPS3 is a ribosome protein in the mitochondria, but not as a component of the mitochondrial 28S subunit. When mitochondrial damages accumulate, RPS3 accumulates in the mitochondria through direct interaction with the cytoplasmic domain of Tom70 to repair damaged DNA^55^. Indeed, we found that knockdown of RPS3A in brown adipocytes decreased the number of mitochondria, as well as the function of mitochondria. The decreased browning of epicardial adipocyte may be due to the decreased expression of RPS3A in EAT, while the decreased browning of paracardial adipocytes may be the secondary phenomenon. Brown fat exerts effects on energy metabolism beyond its role in fuel oxidation. Also, it can serve as a source of endocrine factors^56^. Using co-culture of PVAT-derived CM and endothelial cells, we found the anti-inflammation effect of PVAT on endothelial cells, especially the mature brown adipocytes overexpressing RPS3A. In addition, injection of adenovirus expressing RPS3A shRNA into periaortic adipose tissue damaged the vascular structure and induced inflammation.

Most cohort studies have shown that high blood pressure and increased serum TC increase the risk of developing CAD in men and women in a graded fashion. Besides these factors, there were still many people suffering from CAD without these factors. The results of our study highlight a subpopulation of individuals who display a metabolism disorder signature in their pericardial adipose tissue, which might contribute to their state of coronary artery disease. It is possible that individuals with less brown adipocytes are more at risk for the development of atherosclerosis and CAD. Understanding the mechanism that leads to these differences will equip us with important targets to help stem the tide of such a debilitating disease.

## Materials and methods

### Patients

Fifty-eight patients had angiographic evidence of critical coronary atherosclerosis (CAD) with stenosis greater than 50% in the left main artery or more than 75% in other arteries, involving one, two, or three vessels requiring elective coronary artery bypass grafting (CABG). The second group of 64 control patients without CAD had chronic valvular heart disease with or without stenosis less than 50% in any vessel requiring valve replacement but not CABG. The decision to perform heart surgery was made by the attending cardiologist and cardiac surgeon.

### Fat samples

After the thorax had been opened and before heparinization and cardiopulmonary bypass, fat (0.2 g to 1 g) was obtained from the following sites: EAT over the aortic root; PAT from the inner midcourse and the diaphragm; and thoracic sc adipose tissue (Sub) from the sternotomy incision at the manubrium sterni. Fat samples were trimmed of connective tissue and superficial blood vessels, bisected, and stored separately at −80 °C.

### SYNTAX score

The SYNTAX score II was designed for risk assessment of patients with complex CAD for the purpose of choosing the optimal revascularization method (surgical or percutaneous). It combines the extension and complexity of native CAD with six clinical variables (gender, age, left ventricular ejection fraction, peripheral vascular disease, creatinine clearance, and chronic obstructive pulmonary disease) and was found to be a predictor of 4-year mortality^57^.

### Mice

Male apolipoprotein E deficient (ApoE^−/−^) mice were purchased from the Model Animal Research Center of Nanjing University (Nanjing, Jiangsu, China). Mice were maintained on a normal chow diet (ND) or HFD for 4 months to induce atherosclerosis. For RPS3A knockdown in periaortic adipose tissue assay, 8-week-old male ApoE^−/−^ mice were maintained on a HFD for 1 week before tranverse aortic constriction was performed^58^. Briefly, animals were anesthetized with 2% isoflurane inhalation, intubated, and ventilated with 100% oxygen (0.7 mL/100 g bodyweight, 90/min), and placed in a supine position. A 2-cm median cut along the neck was made and the underlying sternum was revealed. Then a 1.5-cm median hemi-sternotomy was performed, and the prominent thymus was carefully put away to preserve the underlying structures. After isolating the aorta from the pulmonary trunk, a 10-µL Hamilton syringe was placed around the aortic arch to inject adenovirus. Animal handling and experimental procedures were performed following approval from the Institute of Health Sciences Institutional Animal Care and Use Committee.

### Protein and peptide sample preparation

Tissues from CAD and non-CAD patients were lysed by mixing with 150 µL solution containing 4% sodium dodecyl sulfate (SDS), 100 mMTris/HCl pH 7.6, 0.1 M DTT (SDT lysis solution) followed by incubation at 95 °C for 3 min. The DNA was sheared by sonication to reduce the viscosity of the sample. Before sample processing, the lysate was clarified by centrifugation at 16,000×*g* for 5 min. The protein content was determined by measuring tryptophan fluorescence using the Cary Eclipse Fluorescence Spectrometer (Varian, Palo Alto, USA) as previously reported^59^.

The protein samples were digested by sequencing grade modified trypsin (1:50) using the Filter-Aided Sample Preparation protocol^60^. Peptide samples were cleaned of salts and residual oil using STop and Go Extraction tips, dried, and then stored at −80 °C before analysis.

### LC-MS/MS analysis

Each peptide sample was analyzed on the nano HPLC-LTQ-Orbitrap-Veloshybrid mass spectrometer (Thermo Electron Finnigan, San Jose, CA, USA). Peptide mixtures were separated through a nano-emitter column (15 cm length, 75 μM inner diameter) packed in-house with 3 μM C18 ReproSil particles (Dr. Maisch GmbH) and introduced into the mass spectrometer using a nanoelectrospray ion source (source voltage, 1.7–2.2 kV). A linear gradient from 4 to 30% buffer B (buffer A, 0.1% formic acid in ddH2O; buffer B, 0.1% formic acid in acetonitrile) over 120 min was used for peptide separation at a flow rate of 250 nL/min. Collision-induced dissociation (CID) mode was performed on the LTQ-OrbitrapVelosmass spectrometer (Thermo Fisher Scientific, Waltham, MA, USA), and a full scan was acquired at a target value of 1,000,000 ions with resolution *R* = 60,000 at *m*/*z* 400. The top 20 ions were selected at an isolation window of 2.0 *m*/*z* units and accumulated to an AGC target value of 3e4 for tandem mass spectrometry (MS/MS) sequencing. Dynamic exclusion was enabled to void choosing former target ions for 120 s, and lock-mass was enabled using 445.120025. Raw MS data were processed with MaxQuant software version 1.5.2.8 using the default settings with minor changes: oxidation (methionine) and acetylation (protein N-term) were selected as variable modifications, and carbamidomethyl (C) was selected as fixed modification. Database searching was performed using the Andromeda search engine against Swiss-Prot Homo sapiens sequence database (downloaded Nov 2015, 20,196 protein entries), concatenated with known contaminants and reversed sequences of all entries^61^. A false discovery rate of less than 0.01 for proteins and peptides, and a minimum peptide length of 7 amino acids were required.

### Functional analysis

To explore the potential EAT or PAT-associated biological functions between CAD and non-CAD cases, we mapped the differentially expressed proteins into known molecular sets with Kyoto Encyclopedia of Genes and Genomes (KEGG) pathway or Gene Ontology Cellular Component (GO-CC) term annotations. We estimated enrichment significance of specific proteins in each KEGG or GO-CC term based on the hypergeometric test^62^. Therefore, significantly enriched terms were chosen by a *p* value less than 0.05.

### IHC and IF

Adipose tissue obtained was fixed in 4% paraformaldehyde overnight, embedded in paraffin, and cut into 4-mm-thick sections. IHC was performed according to the Vecta Stain Elite ABC kit protocols (Vector Laboratories, Burlingame, CA, USA). Antibody staining was visualized with the enhanced DAB kit (Vector Laboratories). The brown reaction product was quantified using Image-Pro Plus software. For IF staining, mature adipocytes were fixed in 4% paraformaldehyde for 15 min, pre-incubated in blocking buffer (10% normal donkey serum in phosphate-buffered saline (PBS)) for 30 min at room temperature, and incubated sequentially with primary and secondary antibodies diluted in blocking buffer. After washing with PBS, the samples were counterstained with DAPI. Cells were placed on slides using cover slips and mounting medium (SouthernBiotech, Burlingame, CA, USA), and examined under a fluorescence microscope (Nikon, Tokyo, Japan).

### mRNA isolation and assays

Total RNA were extracted by Trizol (Life Technologies, Carlsbad, CA, USA) and stored in DEPC H_2_O at −20 °C. cDNA was synthesized from total RNA with RevertAid First Strand cDNA Synthesis Kit (Thermo Scientific). Real-time PCR was performed using Power SYBR Green qPCR Master Mix (Life Technologies) in 7500 Real Time PCR system (Applied Biosystems, San Francisco, CA, USA). The relative abundance of mRNAs was calculated with 18S mRNA as the invariant control. The primers were from PrimerBank (http://pga.mgh.harvard.edu/primerbank/). Primers are listed in Supplementary Table S2.

### Brown preadipocytes maintenance, differentiation, and treatment

Immortalized brown preadipocytes were generated previously^63^. Briefly, brown preadipocytes were maintained in DMEM medium supplemented with 10% FBS (Gibco, Gaithersburg, MD, USA) and pen/strep, and were cultured in a 5% CO_2_ atmosphere. After seeding onto plates for 24 h, induction cocktail (20 nM insulin and 1 nM T3) was added to the cell culture medium and incubated for 2 days. Then (designated as day 0), differentiation cocktail (20 nM insulin, 0.5 μM dexamethasone, and 0.5 mM 3-isobutyl-1-methyl-xanthine, 125 μM indometacin and 1 nM T3 for 48 h) was added to the cells in fresh media. After 48 h, the media was changed to DMEM supplemented with 10% FBS containing 20 nM insulin and 1 nM T3. The medium was refreshed every other day. Eight days after induction (designated as Day 8), cells was harvested for following analyses.

### Oxygen consumption assays

Oxygen consumption rate (OCR) in EAT, PAT, and SAT was measured using the Seahorse XFe Extracellular Flux Analyzer (Agilent) in a 24-well plate. Adipose tissues (2 mg for EAT and PAT, 4 mg for SAT) were placed into XF24 Islet Capture Microplates. For cultured adipocytes, OCR was measured in 1×10^4^ differentiated adipocytes that were treated with pAd-LacZ shRNA, pAd-RPS3A shRNA, pAd-LacZ, and pAd-RPS3A for 24 h. Cells were subjected to the mitochondrial stress test by adding oligomycin (10 µM for tissue and 1 µM for cells) followed by carbonyl cyanide4-(trifluoromethoxy) phenylhydrazone (FCCP, 9 µM for tissue and 1 µM for cells) and antimycin/rotenone (12 µM for tissue and 1 µM for cells).

### Western blot analysis

Cultured cells or tissues were prepared with the T-PER tissue protein extraction reagent (2% SDS and 60 mM Tris HCl, pH 6.8) with the cocktail of proteinase inhibitors (Roche, Indianapolis, IN, USA) in it. The total protein tissue or cultured cells were homogenized in lysis buffer and were loaded onto the gel (20–40 μg) for electrophoresis. Proteins then were transferred onto 10% SDS-PAGE (sodium dodecylsulfate polyacrylamide gel electrophoresis) and were then transferred to PVDF membranes (Bio-Rad, Hercules, CA, USA). The electroblotted membranes were blocked by TBS containing 5% non-fat milk (Santa Cruz, Dallas, TX, USA) and were probed with primary antibodies overnight at 4°C and immunoblotted with specific antibodies. Primary antibodies against the following proteins were used: UCP1, PGC1α, PRDM16, Cidea (Abcam, Cambridge, UK), PPARγ (CST, Danvers, MA, USA), 422/Ap2 (Santa Cruz Biotechnology), RPS3A (ProteinTech Group, Rosemont, IL, USA), Cyto c (CST), Actin (Sigma, St. Louis, USA).

### Adipocyte-derived CM-dependent vascular inflammation

The mature brown adipocytes were pretreated with adenovirus expressing LacZ or RPS3A for 6 h, after which the medium was replaced. After 12 h, the adipocyte-derived conditioned medium (ACM) was collected. The descending thoracic aorta of mice without PVAT was cut into 2-mm ring segments and suspended in CM for culture, and the medium was replaced every day. After 2 days of culture, the vascular cells were collected for RNA extraction.

### RNA interference

Synthetic siRNA oligonucleotides specific for RPS3A and Crip1 mRNA were designed and synthesized by invitrogen Stealth^TM^ RNAi. The sequence was as follow:

RPS3A Stealth^TM^ RNAi: CCGGAAGAAGATGATGGAAATTT.

Crip1 Stealth^TM^ RNAi: CCCTGCCTGAAGTGCGAGAAATT.

### Adenoviral expression vectors and infection

The adenoviral expression vector pAd/CMV/V5-DEST (Invitrogen, Carlsbad, CA, USA) encoding the RPS3A gene was constructed according to the manufacturer’s protocol. The RPS3A sequence was amplified using the primers 5′-CACCATGGCGGTCGGCAAG-3′ (forward) and 5′-TTACACTGATTCTTGGA-3′ (reverse).

### Statistical analyses

All results are presented as the mean ± SEM. A non-paired Student’s *t* test was used for these analyses. A difference was considered significant at *p < 0.05, **p < 0.01, and ***p < 0.001.

## Electronic supplementary material

supplemental material

## Electronic supplementary material

**Supplementary Information** accompanies the paper at (10.1038/s41421-018-0041-2).
