# Supplementary material for: RPS3A positively regulates the mitochondrial function of human periaortic adipose tissue and is associated with coronary artery diseases
Source: Cell Discov. 2018 Aug 21;4:52. doi: 10.1038/s41421-018-0041-2 (PMC6102269; doi:10.1038/s41421-018-0041-2)
Supplement: Supplementary file 1 — supplemental material [file 41421_2018_41_MOESM1_ESM.docx]

**Supplementary Table S1: clinical characteristics of the two study groups.**

|  | **Non-CAD** | **CAD** | **P value** |
| --- | --- | --- | --- |
| **Participants, n** | 64 | 58 |  |
| **Age, y** | 55.14±1.51 | 63.21±2.54 | **P<0.0001** |
| **Sex, (M/F)** | 28/36 | 40/18 | **P=0.005** |
| **BMI, kg/m2** | 26.32±0.44 | 27.15±1.19 | P=0.1802 |
| **Waist, cm** | 94.19±2.75 | 99.63±3.63 | **P=0.0048** |
| **Smoking, n (%)** | 10.94 | 13.79 | P=0.731 |
| **Hypertension, n (%)** | 15.62 | 51.72 | **P<0.0001** |
| **Hyperlipidemia, n (%)** | 26.56 | 17.24 | P=0.091 |
| **Diabetes mellitus, n (%)** | 28.12 | 24.13 | P=0.617 |
| **Cholesterol, mmol/L** | 4.15±0.49 | 4.30±0.64 | P=0.4947 |
| **Triglycerides, mmol/L** | 1.46±0.3 | 1.94±0.38 | P=0.1161 |
| **HDL, mmol/L** | 1.18±0.17 | 1.09±0.07 | P=0.9635 |
| **LDL, mmol/L** | 2.33±0.33 | 2.54±0.44 | P=0.3974 |

**Supplementary Table S2: Primers for real-time qPCR used in this study.**

|  | **Forward** | **Reverse** |
| --- | --- | --- |
| **Human UCP-1** | AGGTCCAAGGTGAATGCCC | TTACCACAGCGGTGATTGTTC |
| **Human PGC1α** | TCTGAGTCTGTATGGAGTGACAT | CCAAGTCGTTCACATCTAGTTCA |
| **Human Cidea** | GATGCCCTCGTCATCGCTAC | GCGTGTTGTCTCCCAAGGTC |
| **Human PRDM16** | CTTCGGATGGGAGCAAATACTG | TCCACGCAGAACTTCTCACTG |
| **Human C/EBPα** | TGATGCCGTTCACACACTTCA | CAAGGCCATAAGGCACTGC |
| **Human PPARγ** | ACCAAAGTGCAATCAAAGTGGA | ATGAGGGAGTTGGAAGGCTCT |
| **Human Ap2** | ACTGGGCCAGGAATTTGACG | CTCGTGGAAGTGACGCCTT |
| **Human CS** | TGCTTCCTCCACGAATTTGAAA | CCACCATACATCATGTCCACAG |
| **Human OGDH** | GGCTTCCCAGACTGTTAAGAC | GCAGAATAGCACCGAATCTGTTG |
| **Human CYCS** | CTTTGGGCGGAAGACAGGTC | TTATTGGCGGCTGTGTAAGAG |
| **Human FASN** | AAGGACCTGTCTAGGTTTGATGC | TGGCTTCATAGGTGACTTCCA |
| **Human DBI** | CAGAGGAGGTTAGGCACCTTA | TATGTCGCCCACAGTTGCTTG |
| **Human GLIPR2** | GAACCTTGCATGGGCATCCTA | GGCTGCTGGAAGTTATAGTTCTT |
| **Human TST** | GACTGGACTCGGGCCATATC | ACGTGGCAATGAGAGGCTG |
| **Human IGHM** | ACCGTGTCCGAAGAGGAATG | CGGTGGTACTGTAGAAGAGGC |
| **Human RPS3A** | TGGATCTTACCCGTGACAAAATG | TGACATCAACGTGAGCTTCAATC |
| **Human HSD17B12** | TGTCCCACTCTTGACCATCTAT | CTTGCTCCTATACTCCTCATGGA |
| **Human Crip1** | CCTGCCTGAAGTGCGAGAAAT | CCTTTAGGCCCAAACATGGC |
| **Human Col1a1** | GTGCGATGACGTGATCTGTGA | CGGTGGTTTCTTGGTCGGT |
| **Human LCN2** | GACAACCAATTCCAGGGGAAG | GCATACATCTTTTGCGGGTCT |
| **Human ARβ** | GACCAACGTGTTCGTGACTTC | GCACAGGGTTTCGATGCTG |
| **Human ThRβ** | TGATGATGTGAACGACCAGAGT | AGTAACTGGGGATGTACCCTTT |
| **Human TNFα** | GAGGCCAAGCCCTGGTATG | CGGGCCGATTGATCTCAGC |
| **Human Mrc1** | GGGTTGCTATCACTCTCTATGC | TTTCTTGTCTGTTGCCGTAGTT |
| **Human 18s** | GTAACCCGTTGAACCCCATT | CCATCCAATCGGTAGTAGCG |
| **Mouse RPS3A** | AACAAGCGCCTGACGAAAG | AGTGTCTTCCCGATGTTCCTAAT |
| **Mouse Crip1** | AAGTGCGACAAGGAGGTGTAT | AGAGGTCAGTGTCTTTCCACATT |
| **Mouse TNFα** | GACGTGGAACTGGCAGAAGAG | ACCGCCTGGAGTTCTGGAA |
| **Mouse IL-1β** | GCAACTGTTCCTGAACTCAACT | ATCTTTTGGGGTCCGTCAACT |
| **Mouse IL-6** | CCACGGCCTTCCCTACTTC | TTGGGAGTGGTATCCTCTGTGA |
| **Mouse ICAM1** | TGCCTCTGAAGCTCGGATATAC | TCTGTCGAACTCCTCAGTCAC |
| **Mouse VCAM1** | AGTTGGGGATTCGGTTGTTCT | CCCCTCATTCCTTACCACCC |
| **Mouse 18s** | CGCCGCTAGAGGTGAAATTCT | CATTCTTGGCA1AATGCTTTCG |

**
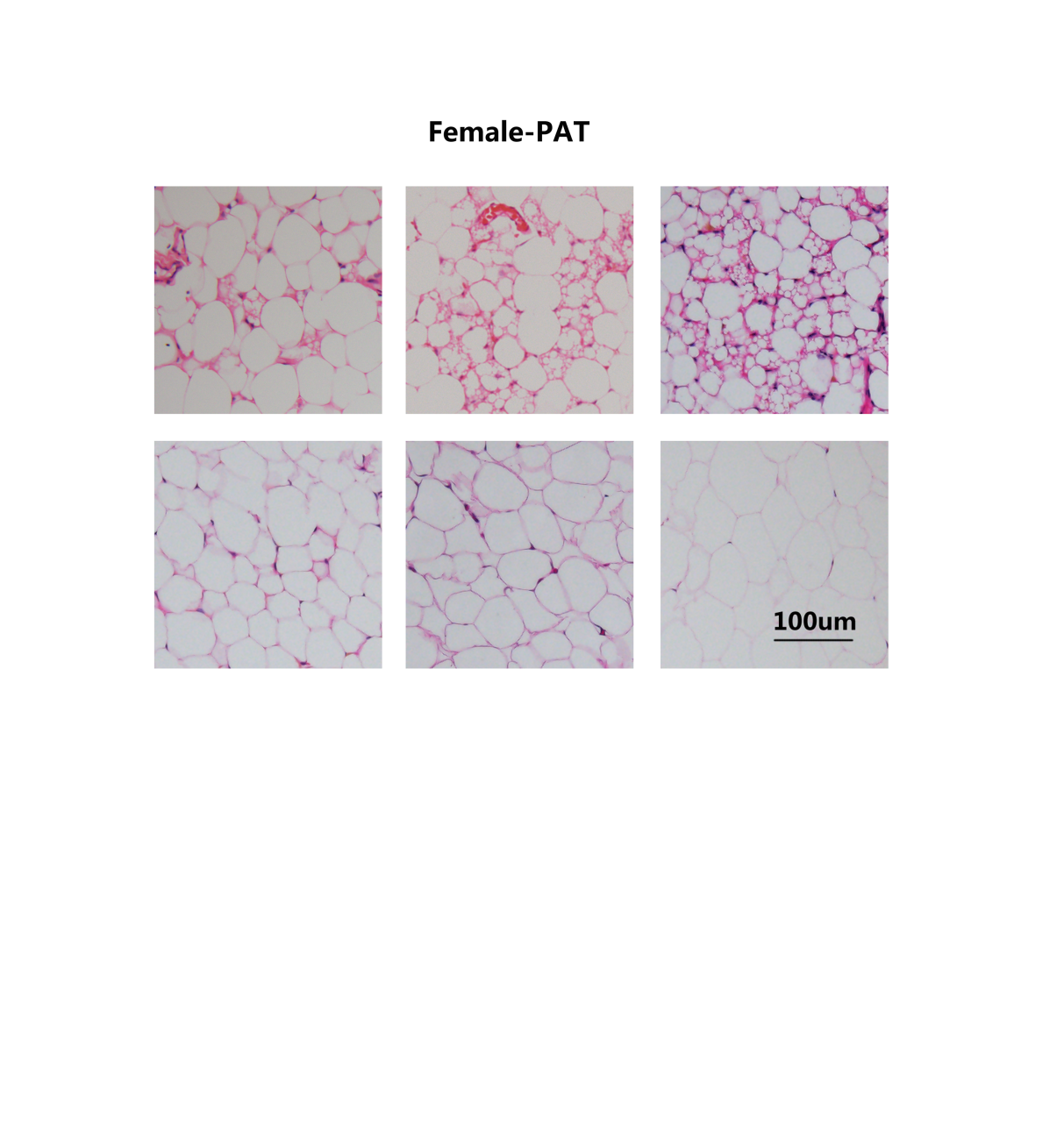
**

**Supplementary Figure 1.** Hematoxylin-and-eosin staining of sections obtained from human female paracardial adipose tissue (PAT).


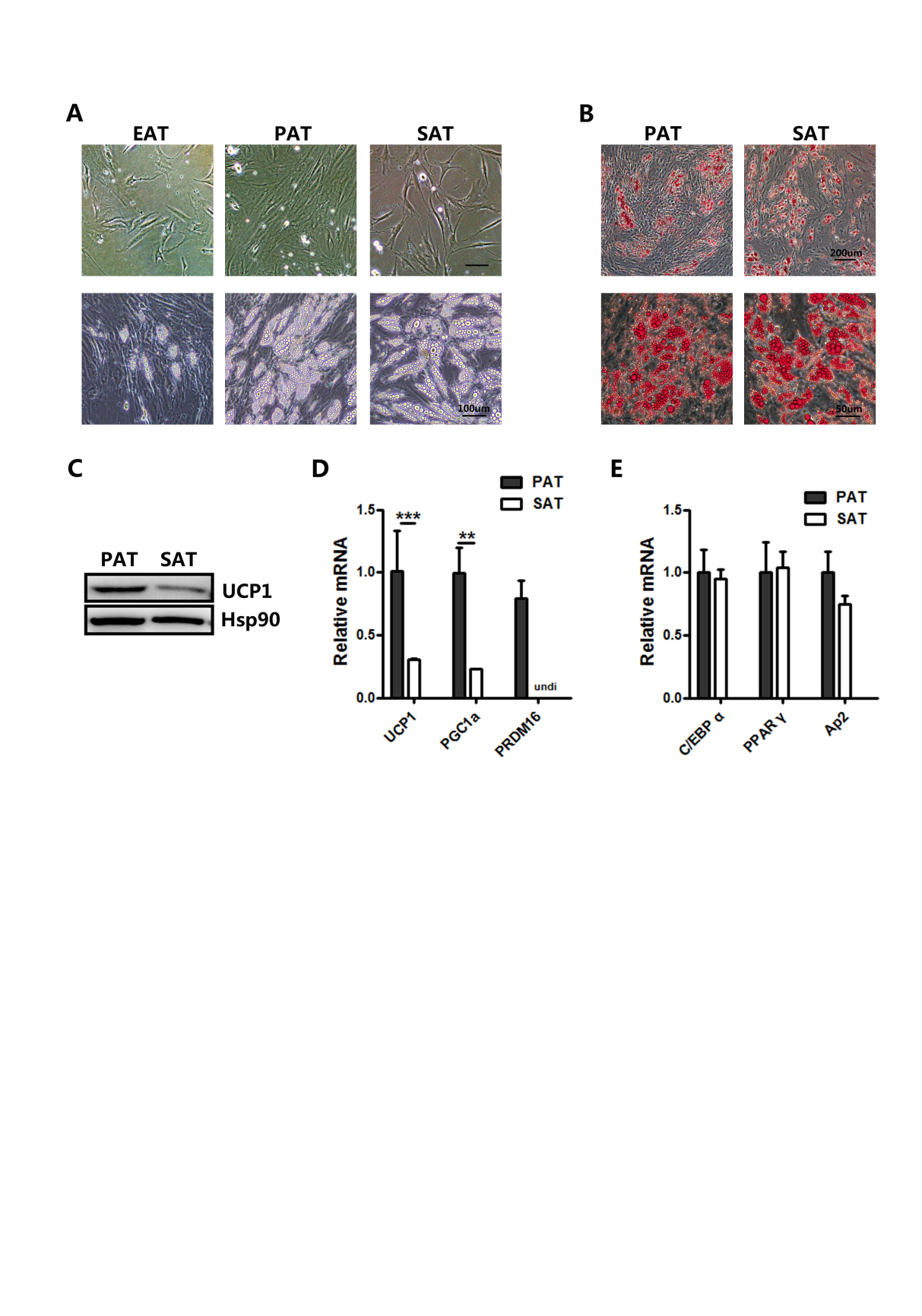


**Supplementary Figure 2. Brown adipocyte differentiation of stem cell from EAT, PAT and SAT.**

a, Morphology of cultured stem cells (up) and differentiated mature adipocyte (bottom) from human EAT, PAT and SAT. Scale bar = 200μm.

b, Oil Red O staining of adipocytes differentiated from human PAT and SAT on day 8. Scale bar = 200μm (up), Scale bar = 50μm (bottom).

c, Western blot experiment using antibodies against UCP1 of mature adipocyte differentiated from human PAT and SAT.

d & e, Real-time PCR data showing the fold induction of indicated brown (D) and white (E) adipocyte related genes with expression normalized to the housekeeping gene 18s in mature adipocyte differentiated from human PAT and SAT (n=3).


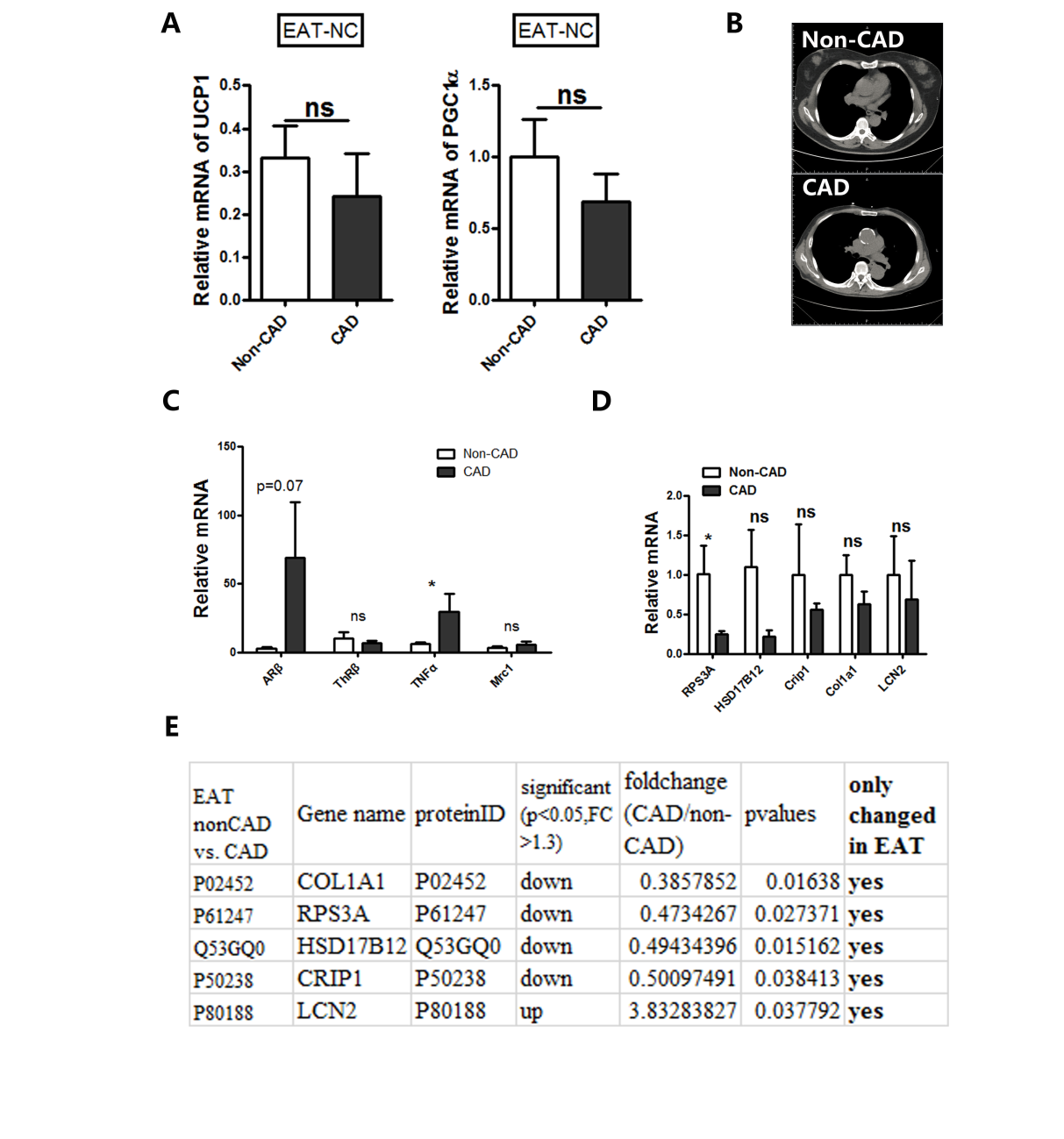


**Supplementary Figure 3. The lesion of periaortic adipose tissue is associated with CAD.**

a, Real-time PCR data showing the fold induction of indicated brown adipocyte related genes with expression normalized to the housekeeping gene 18s in normal EAT from patients with or without CAD (n=12-18).

b, Computed tomography angiography image shows a calcified soft plaque (arrow) in the artery root from CAD patients.

c, Real-time PCR data showing the fold induction of indicated browning induction genes with expression normalized to the housekeeping gene 18s in periaortic EAT from patients with or without CAD (n=12-18).

d, Reat-time PCR validation of genes selected from the EAT proteomic analysis of patients with or without CAD (n=7-9).

e, Proteins changed significantly in EAT from proteomic analysis.

**
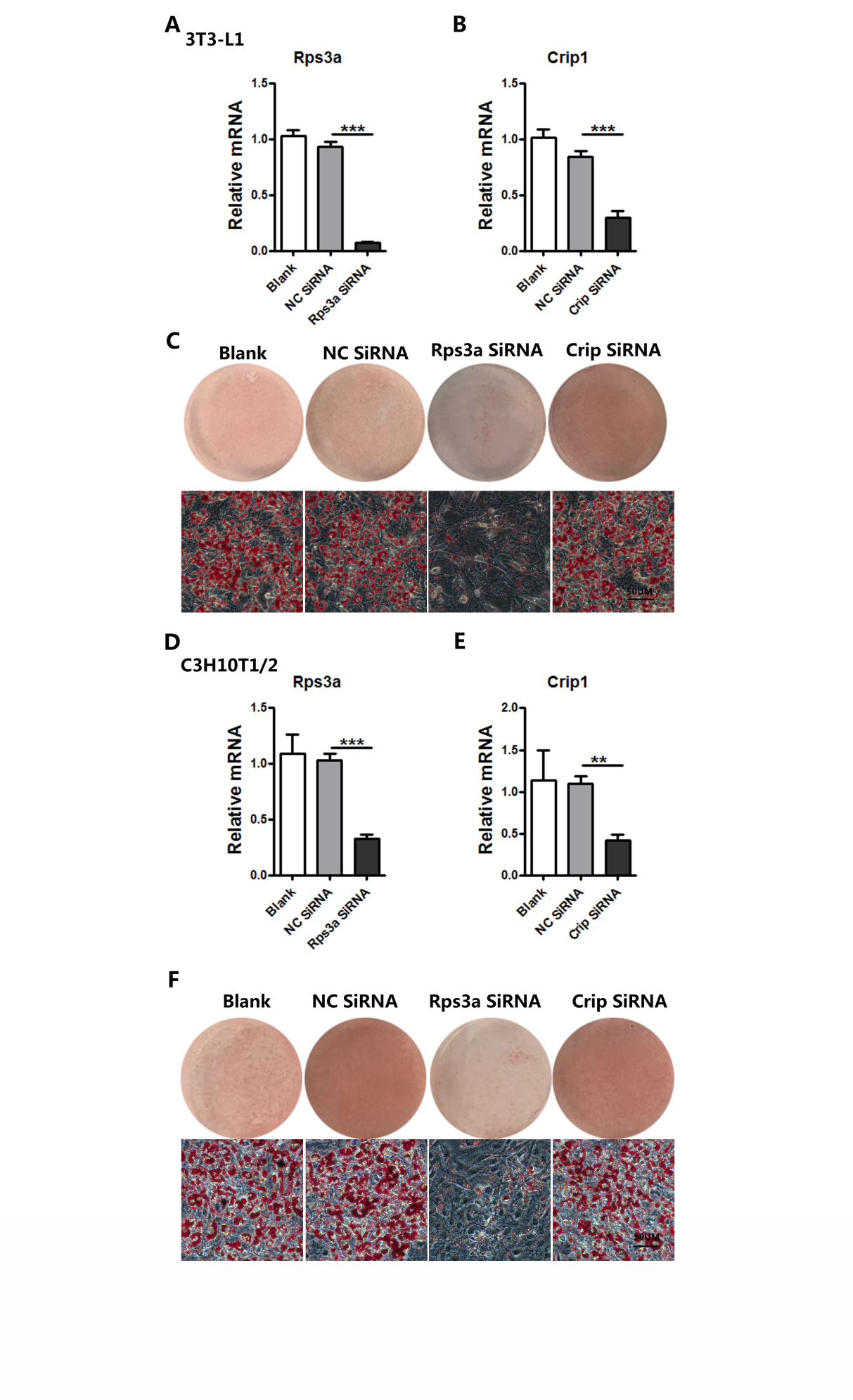
**

**Supplementary Figure 4. Downregulation of RPS3A inhibits adipogenic differentiation.**

a-c, 3T3-L1 cells were treated with RNAi before adipogenic induction and collected for Q-PCR to determine relative mRNA expression levels of RPS3A (A, n=3) and Crip1 (B, n=3), Oil Red O staining of adipocytes differentiated from 3T3-L1 on Day 8 (C).

d-f, C3H10T1/2 cells were treated with RNAi before adipogenic induction and collected for Q-PCR to determine relative mRNA expression levels of RPS3A (D, n=3) and Crip1 (E, n=3), Oil Red O staining of adipocytes differentiated from C3H10T1/2 on Day 8 (F).

**
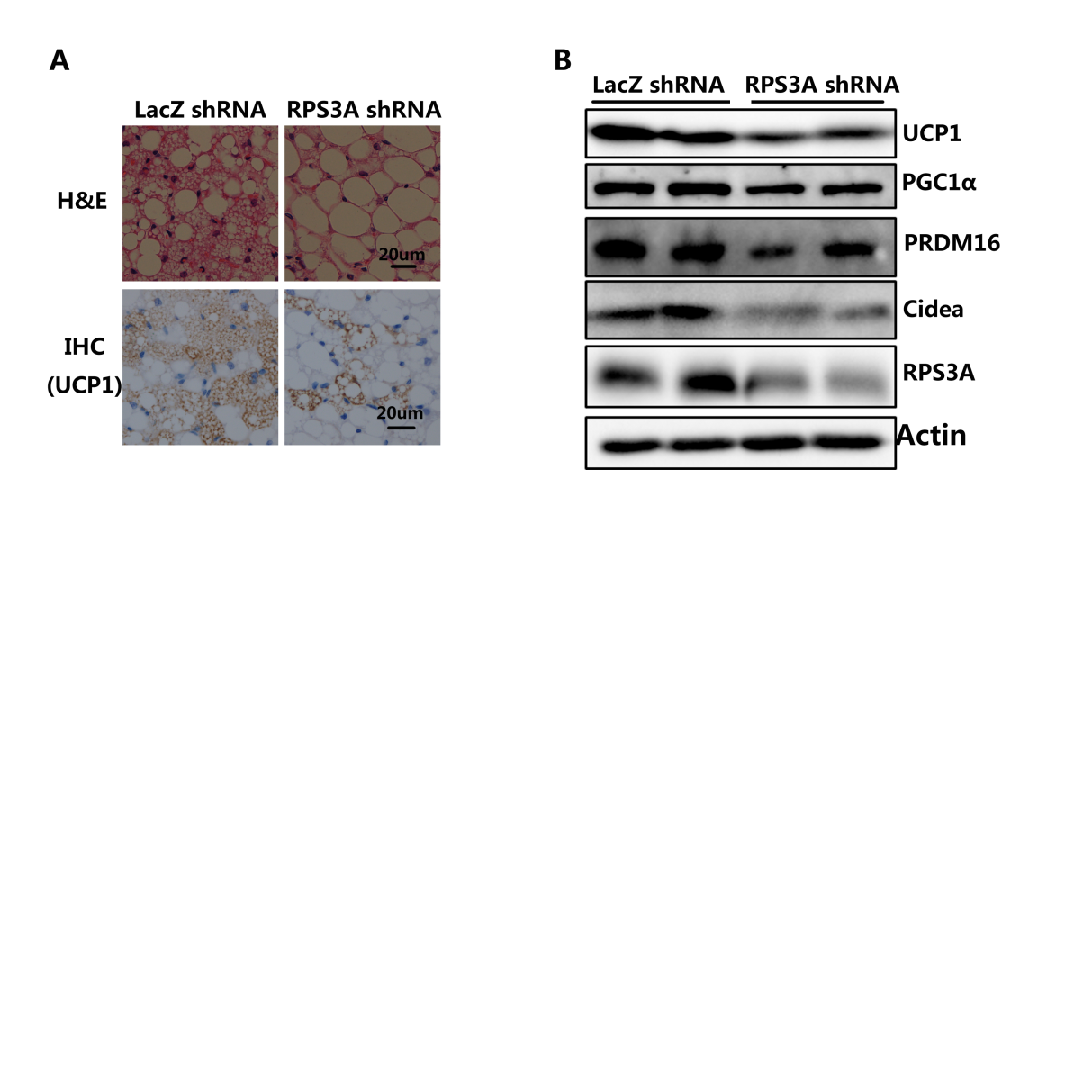
**

**Supplementary Figure 5. RPS3A is required for browning in subcutaneous adipose tissue.**

Adenovirus expressing RPS3A shRNA was injected twice a week s.c. adjacent to one side of the inguinal fat pad, and LacZ shRNA was injected at the contralateral site as a control for 4 weeks beginning at age 2 month. Then the mice was exposed in cold environment for 24hr.

a, H&E and IHC-UCP1 staining of the inguinal WAT fat pad treated with RPS3A shRNA or LacZ shRNA.

b, Western blot analyzing brown related proteins from the inguinal WAT fat pad after treatment with RPS3A shRNA or LacZ shRNA.
